# Relationship between Nutritional Risk, Clinical and Demographic Characteristics, and Pressure Ulcers in Patients with Severe Acquired Brain Injuries Attending a Rehabilitative Program

**DOI:** 10.3390/nu15153336

**Published:** 2023-07-27

**Authors:** Chiara Francesca Gheri, Luca Scalfi, Barbara Biffi, Silvia Pancani, Sara Madiai, Olivia Di Vincenzo, Michele Ghaderi, Rebecca Celoni, Mara Dalladonna, Francesca Draghi, Daniela Maccanti, Claudio Macchi, Anna Maria Romoli, Francesca Cecchi, Bahia Hakiki, Maria Luisa Eliana Luisi

**Affiliations:** 1 IRCCS Fondazione Don Carlo Gnocchi ONLUS Firenze, 50143 Florence, Italyspancani@dongnocchi.it (S.P.); mghaderi@dongnocchi.it (M.G.); rceloni@dongnocchi.it (R.C.); mdalladonna@dongnocchi.it (M.D.); fdraghi@dongnocchi.it (F.D.); dmaccanti@dongnocchi.it (D.M.); cmacchi@dongnocchi.it (C.M.); amromoli@dongnocchi.it (A.M.R.); fcecchi@dongnocchi.it (F.C.); bhakiki@dongnocchi.it (B.H.); mluisi@dongnocchi.it (M.L.E.L.); 2 Human Nutrition and Dietetics, Department of Public Health, Federico II University, 80131 Naples, Italy; scalfi@unina.it (L.S.); olivia.divincenzo@unina.it (O.D.V.); 3 Santa Maria del Pozzo Private Hospital, Somma Vesuviana, 80049 Naples, Italy; 4 Department of Experimental and Clinical Medicine, University of Florence, Largo Brambilla, 50134 Florence, Italy

**Keywords:** pressure ulcer, nutrition, MUST, CONUT, rehabilitation, severe acquired brain injury, stroke

## Abstract

Preliminary evidence in the literature suggests a high prevalence of malnutrition (undernutrition) in patients with severe acquired brain injuries (sABI), with an expected negative impact on clinical outcomes and pressure ulcers (PUs) in particular. In a retrospective cohort study on patients discharged from intensive care units (ICU) and admitted to an intensive rehabilitation unit (IRU), the risk of malnutrition was systematically assessed, in addition to standard clinical procedures (including PUs evaluation), using two different tools: the Malnutrition Universal Screening Tool (MUST) and the Controlling Nutritional Status (CONUT) tool. Eighty-eight patients were included in the analysis. A high proportion (79.5%) of patients with sABI suffered from PUs, being older and more frequently men, with a longer ICU stay between the event and admission to IRU, and a greater MUST score. At discharge, when compared to patients whose PUs had healed, those with persisting PUs were more often men and had the worst cognitive performance at admission. As for nutritional risk, the baseline CONUT score was identified as an independent negative predictor of PUs at discharge by the logistic regression model. In conclusion, the assessment of nutritional risk using simple standard tools may be useful in the clinical evaluation of sABI patients with PUs.

## 1. Introduction

Severe acquired brain injuries (sABI) include several neurological conditions due to traumatic, post-anoxic, hemorrhagic, and ischemic injuries that cause a coma for at least 24 h [1].

The worldwide prevalence of sABI is high. In Italy, the sABI incidence rate is estimated between 10 and 15 new cases per 100,000 persons per year and the prevalence rate is estimated between 300 and 800 cases per 100,000 persons per year [2].

sABI damage can cause many impairments, leading to severe, chronic, and often permanent sensory, motor, cognitive, and/or behavioral disability. Disability caused by sABI has a significant impact on patients’ families and healthcare systems. Patients who survived sABI need long-term management; it is necessary for a comprehensive multidimensional assessment of these patients to tailor a patient’s individual rehabilitation plan and to support the patient’s family [1].

Patients were transferred to sABI rehabilitation wards from intensive care units (ICUs), a setting with recurrent clinical complications due to the instability, vulnerability and heterogeneity of patients [3,4]; of note is that pressure ulcers (PUs) are one of the adverse events with a higher prevalence and incidence [5]. Critical ill patients with PUs have a poor quality of life, an increased length of hospitalization, and high morbidity and mortality [6]; the mortality rate is five times greater in patients with PUs compared to those without, which is about 64% [7]. In addition, the management of PUs is a considerable cost for the health system, mainly due to the additional nursing time [6,8].

A high risk of PUs is also reported in patients with sABI [7] due to hypermetabolic and hypercatabolic stress responses, in addition to the impairment in cognitive function, mobility and sensory perception, food swallowing and chewing, and bowel and bladder incontinence [9,10].

Previous data have shown that PU onset in patients with sABI is significantly associated with mortality at 21 days and with recovery status at 3 months [3,10]. 

In addition, few recent reports concerning patients with moderate to severe acquired brain injuries have indicated that malnutrition was common at admission to the intensive rehabilitation unit (IRU) after hospitalization in the intensive care unit (ICU) [11,12]. Thus, an early assessment of nutritional risk is crucial to carry out the needed therapies and to prevent the consequences.

It has been described as a vicious circle between malnutrition and PU onset [13]. Malnutrition increased the risk of inflammation, a well-known risk factor for PU onset; PUs perpetuate inflammation and increase the infection risk which causes poor nutritional status, causing tissue protein breakdown and body wasting. 

Among sABI patients, PU onset can affect recovery from brain injury [14,15]. PUs are associated not only with higher morbidity and mortality but also with many secondary negative consequences such as discomfort and pain [14]. After sABI, some patients recover full consciousness, but they may still present lifelong severe cognitive impairment; on the other hand, some patients may survive in a state of prolonged disorder of consciousness [2].

Among these patients with impaired consciousness or impaired cognitive status, the possible perception of suffering cannot be excluded, so it is a priority to prevent PU onset [14].

Taking into account the multifactorial etiology of PUs, malnutrition (from here onwards, this term refers to undernutrition) is a preventable PU risk factor to be considered with great interest [16] due to the fact that a significant proportion of hospitalized patients are malnourished, especially the elderly [14], with a higher prevalence in the ICU (>60% at hospital admission) [4].

Proper and specific nutrition is important not only to prevent but also to treat PU.

Macro- and micronutrients in specific amounts have a key role in promoting the growth, maintenance, and repair of body tissues [17]. Skin viability and wound healing are sustained by a positive energy and protein intake; for malnourished or at-risk patients with PU, the EPUAP recognizes the role of supplements and recommends supplements with micronutrients enriched with arginine, zinc, and antioxidants [17,18,19].

A systematic approach to addressing malnutrition in hospitalized patients should consider assessing the nutritional risk at admission [20]. The American (ASPEN) and European (ESPEN) Societies of Parenteral and Enteral Nutrition give an almost identical definition of nutritional screening: “a process to identify an individual who is malnourished, or at risk of malnutrition, to determine if a detailed nutritional assessment is required” [20].

Several tools/procedures have been proposed for nutritional screening [20] before a more detailed procedure is carried out for the assessment of nutritional status to diagnose and quantify the type and degree of malnutrition in higher-risk patients (Nutrition Care Process). Screening tools must be easily sensitive, specific, and reproducible. Commonly, they include multiple items regarding unplanned weight loss and nutritional intake assessment, patient’s functional capacity, and disease-associated metabolic stress evaluation [20]. Others include basic laboratory tests, such as albumin levels that are possibly associated with inflammatory status and clinical outcomes.

In the literature, to our knowledge, there is little to no information on the evaluation of nutritional risk (i.e., the risk of undernutrition) in patients with sABI and PUs admitted to rehabilitation units.

In the present paper, two tools for nutritional screening were considered: the Malnutrition Universal Screening Tool (MUST) [21] and the Controlling Nutritional Status (CONUT) score [22]. As recognized by ESPEN, the MUST tool [21] (expected values 0–6) may be used in different settings such as hospitals, elderly care, and community; risk levels are defined based on BMI, unintentional weight loss in the previous 3–6 months, and the presence of an acute disease plus a reduction in food intake > 5 days [20]. The CONUT score (expected values 0–12) was proposed in particular for hospitalized patients [22] and is calculated taking into account serum/plasma albumin and cholesterol as well as blood lymphocyte count [20]; it is considered a nutritional inflammation index, first of all, due to the impact of inflammation on albumin concentration.

CONUT score, being based on easy-to-obtain measures, can be adopted in many patients. Moreover, the CONUT score can be used as a permanent screening system [22]. 

Evidence reports that the CONUT score is an independent prognostic factor in patients with chronic heart failure, myocardial infarction, and peripheral arterial disease [23].

Recently, it was observed that a high CONUT score is significantly negatively associated with complete wound healing in patients with critical limb ischemia, ischemic ulcer or gangrene [23].

In this framework, the aim of the present study was to evaluate clinical and demographic characteristics as well as nutritional risk in sABI patients admitted with and without PU to an intensive rehabilitation unit (IRU). A major objective is that the association of nutritional risk with the presence of PUs at discharge has been evaluated.

## 2. Materials and Methods

### 2.1. Study Setting and Design

We conducted a non-concurrent observational retrospective monocentric study. Subjects were selected from a database of patients with sABI admitted to IRU of the IRCCS Fondazione Don Carlo Gnocchi of Florence from May 2015 to April 2016. Unfortunately, there were no data available on long-term follow-up. Indeed, we planned a more extensive study including long-term follow-up. Sadly, organizing this second-line study proved to be extremely difficult due to the problematic clinical conditions of these patients.

The legal surrogate of all patients signed a written informed consent. The Ethics Committee of the IRCCS Fondazione Don Carlo Gnocchi ONLUS approved the study (approval number 08_15/04/2015); the study was carried out in agreement with the principles of the Helsinki Declaration.

Fondazione Don Carlo Gnocchi benefits from direct public coverage and patients with sABI were transferred to its intensive rehabilitation units (IRUs) from intensive care units (ICUs) of the National Health Care System, for the purpose of attending rehabilitation sessions; all patients attend the rehabilitation sessions throughout the hospitalization time.

In our study, the variable “time post event” refers to the time between the event and admission to the intensive rehabilitation unit in those patients with severe acquired brain injury corresponding to the ICU stay.

### 2.2. Study Population

The files of patients with sABI were retrospectively analyzed for dates and the data were collected by nutritional service operators. Inclusion criteria were admission to the IRCCS Fondazione Don Carlo Gnocchi after a sABI and age 18+. All patients were hemodynamically stable. The lack of a legal guardian was the only exclusion criterion.

There were no alcoholic patients or patients with dementia/cognitive impairment or other types of brain damage before the event that caused the severe acquired brain lesion.

### 2.3. Clinical Evaluation

During the hospitalization, all patients attended a comprehensive multidisciplinary rehabilitation program according to their clinical needs [24].

A dietitian predicted energy and protein requirements using the weight-adjusted Harris–Benedict equation and provided a tailored nutritional plan according to the patient’s personal and clinical needs. A daily protein intake (1.2–1.5 g/kg body weight) and energy intake (30–35 kcal/kg body weight), according to international guidelines, was established [19,25], providing a lower protein intake for patients with acute renal failure (0.8 g/kg body weight) and a higher intake in case of sepsis. Depending on the patient’s comorbidities and goals, an adequate fluid intake for hydration was provided daily [19].

All patients with PUs were supplemented with a disease-specific enteral formula enriched with protein and high in arginine, zinc, and antioxidants, i.e., vitamin C and selenium [17].

According to current Italian recommendations for the evaluation of patients with sABI [1], PUs were defined by the European Pressure Ulcer Advisory Panel (EPUAP) description [19] as a local skin injury and/or a subcutaneous injury on a bony prominence occurring with pressure or friction with pressure.

Within one week after IRU admission, skilled operators assessed the clinical and functional conditions of the patients including the consciousness and cognitive status using, respectively, the Italian version of the Coma Recovery Scale-revised (CRS-R) and the Level of Cognitive Functioning Scale (LCF) score; the functional autonomy using the Functional Independence Measure (FIM) score. Dysphagia was assessed by the oral feeding recovery and severity of dysphagia using the Functional Oral Intake Scale (FOIS) score and the disability using the Disability Rating Scale (DRS) score.

Skilled nursing professionals assessed the PU grade, repeated the PU evaluation during the hospital stay, and provided PU prophylaxis and treatment according to international guidelines [19].

According to international guidelines, all patients with PUs received similar treatments, and nurses applied topical treatments depending on the type and grade of the lesion. 

All clinical and functional evaluations were repeated by the same skilled operators at the discharge.

### 2.4. Nutritional Risk and Anthropometric Measures Assessment

Patients’ energy intake was collected from the dietetics record by the dietitian. 

It was not possible to obtain accurate information on patients’ diets before the sABI due to the complex clinical conditions of these patients.

Patients’ legal surrogates provided information on patients’ diets and all the patients included in the analysis had a Mediterranean-like diet pattern, typical of Tuscany.

The Fondazione Don Gnocchi’s nursing team performed the malnutrition risk screening with a validated screening tool, the Malnutrition Universal Screening Tool (MUST) [21], within 24 h from admission.

MUST is a commonly used tool to identify patients who are at risk of malnutrition, recognized by ESPEN to be used in different settings (hospitals, elderly care, and in the community) by all care workers. In bedridden patients, body weight, expressed in kg, was measured by a lifter, with patients wearing light clothes. Height was estimated by measuring the length of the left (preferable, if possible) forearm (ulna) between the elbow (olecranon process) and the midpoint of the styloid process (wrist’s prominent bone) [21].

The nursing team also registered the percentage of unplanned weight loss and starvation (if is likely that there has not been or there will be no nutritional intake for >5 days) to obtain, together with BMI, the MUST score: a score = 0 means a low risk, a score = 1 a medium risk, and score ≥ 2 a high risk of malnutrition.

### 2.5. Biochemical Data, Nutritional Risk, and Inflammatory Status Assessment

Venous blood was collected in the morning.

Laboratory data including serum albumin, total cholesterol level, and total lymphocyte count were considered to assess the nutritional status by the validated Controlling Nutritional Status (CONUT) screening tool [22].

Neutrophil and lymphocyte counts were also considered to calculate the neutrophil/lymphocyte ratio (NLR). NLR is routinely used in clinical wards to evaluate systemic inflammation. The NLR is calculated using the absolute neutrophil count divided by the absolute lymphocyte count [26].

We registered blood samples collected at admission to the IRU and discharge. It was possible to retrieve coherent data for all patients only at baseline and discharge. Subsequently, laboratory tests were done for clinical purposes differently between patients. There were no data available on long-term follow-up after discharge from the IRU.

### 2.6. Demographical and Clinical Data

For each patient, the following data were registered at admission and discharge: sex; age; sABI etiology (traumatic/hemorrhagic/ischemic/anoxic/other such as carbon monoxide poisoning or encephalitis); time post event (in days); grade of PU; total daily calories; CRS-R total score; LCF score; FIM score; FOIS score; DRS score; MUST score; and CONUT score.

For each patient, the length of stay (in days) in the IRU was also registered.

Data were extracted from a database of measures recorded during patients’ hospital stays.

### 2.7. Statistical Analysis

Statistical analyses were performed using SPSS v28 (IBM, Armonk, NY, USA). Categorical and dichotomous variables were summarized using frequencies and percentages, and continuous variables using median and interquartile range [IQR] due to the non-normality of the distributions verified using the Shapiro–Wilk test.

Demographic and clinical characteristics were compared between patients with PU at admission and those without, using the Mann–Whitney U test for continuous variables and the chi-square test for categorical and dichotomous variables. The same tests were used to compare clinical and demographic characteristics between patients who completely healed their PU during their IRU stay and those who did not. Variables found to be significantly different between the two groups were included as independent variables in a logistic regression model. The presence of PU at discharge was assumed as the dependent variable. In all analyses, a *p*-value < 0.05 was considered significant.

## 3. Results

### 3.1. Characteristics of Patients with and without PU at Admission

**Figure 1:**
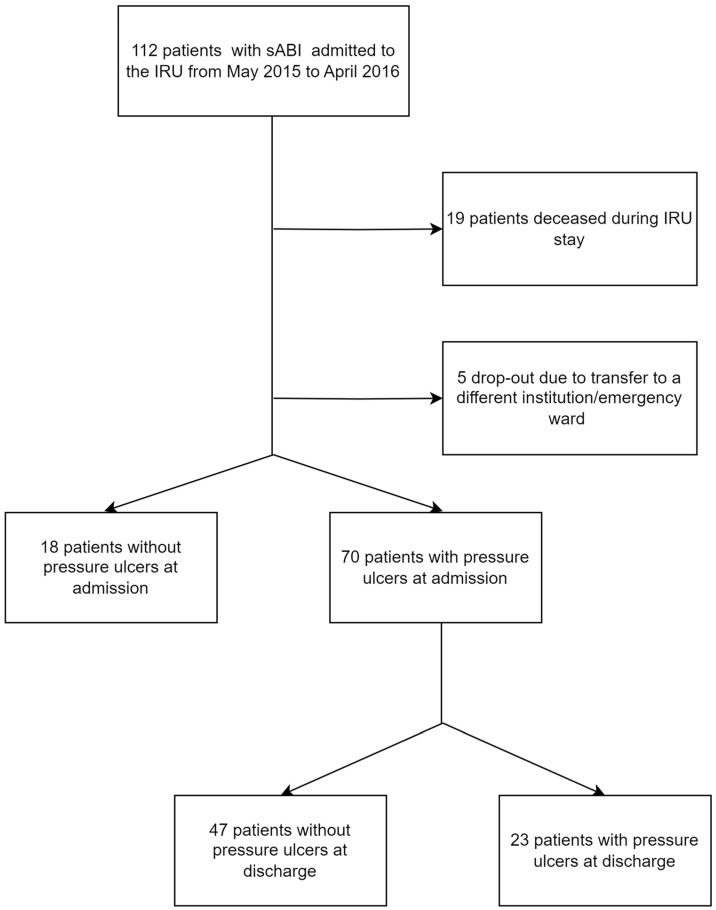
Flowchart.

During the study period, 112 patients admitted to the IRU of the IRCCS Fondazione Don Carlo Gnocchi of Florence from May 2015 to April 2016 following a sABI, were evaluated (Figure 1).

Out of the 88 patients included in the analysis, 70 (80%) presented with PU at admission. The characteristics of patients with and without PU at admission are summarized in Table 1. A chi-square test was used to evaluate differences in the distribution of etiologies between patients with/without pressure ulcers at admission. No significant difference was found between the distributions recorded in the two groups (*p* = 0.637).

Patients admitted with PU had a median age of 64 [IQR 20] years and 34% were women. Compared to patients without PU at admission, those with PU had a significantly longer time post event, i.e., the time between the event and admission to IRU (median value 44 vs. 30, *p* = 0.034), during which patients were hospitalized in the ICU; in addition, patients with PU had a higher MUST score (*p* = 0.008), with no difference in CONUT score. The prevalence of a moderate–severe nutritional risk (MUST ≥ 2 and CONUT ≥ 5) in patients with PU at admission was 50.7% while in patients without PU at admission was 22.2%.

### 3.2. Characteristics of Patients with and without PU at Discharge

At discharge, 23 patients (33%) still presented with PU. A chi-square test was used to evaluate differences in the distribution of etiologies between patients with/without pressure ulcers at discharge. No significant difference was found between the distributions recorded in the two groups (*p* = 0.732).

The comparison between the patients who had their ulcers completely healed during the IRU stay and those who had not is shown in Table 2.

The latest group had a lower percentage of women (13.0% vs. 44.7%, *p* = 0.009), lower LCF at admission (median value 2 vs. 3, *p* = 0.042), higher ulcers severity (median PU grade 3 vs. 2, *p* = 0.005), and a higher CONUT score at admission (median value 6 vs. 4, *p* = 0.039). The prevalence of moderate–severe nutritional risk was much greater in the patients who still presented with PU.

### 3.3. Association between the Presence of PU at Discharge and Clinical Characteristics at Admission

A logistic regression model was built to investigate the relationships of PUs at discharge with demographic and clinical characteristics at admission: sex, level of cognitive function (LCF) at admission, ulcer severity (higher grade), and the CONUT score at admission. According to the results shown in Table 3, only ulcer severity (OR = 2.060, *p* = 0.049) and the CONUT score (OR = 1.366, *p* = 0.032) were significantly associated with an increased likelihood of having PU at discharge.

## 4. Discussion

This study has explored demographic and clinical characteristics as well as nutritional risk in patients with sABI with or without PUs admitted to IRU, evaluating the variables associated with PU at admission and those related to PU healing. As a new finding, nutritional risk assessed using the CONUT score emerged as a risk factor for the presence of PUs at discharge in patients with sABI.

To our knowledge, the prognostic role of the CONUT score with regard to pressure ulcer healing in sABI patients has not yet been examined. Currently, there are few studies investigating the prognostic role of nutritional parameters in sABI patients, and a lack of studies on the factors that contribute to PU onset in these patients [14].

At admission to IRU, a high proportion (79.5%) of patients with sABI suffered from PU (Table 1) in agreement with the notion that they may have developed severe complications during previous hospital stay [7].

Patients admitted with PU were more frequently men (66%) and older (median age of 64 years) compared to those admitted without PU. Contrasting findings have been produced on the relationships between the male sex and PU [27,28]; nevertheless, the Waterlow pressure ulcer risk assessment scale considers sex as an independent risk factor on PU onset [29] and previous observations reported more significant results indicating male sex as a risk factor [30].

As for age, a recent meta-analysis reported a consistent association not only with the onset of new PU but also with the worsening of prior lesions [27]. Patients over 60 years have a greater risk compared to the younger ones due to factors such as comorbidities, reduced mobility, and pain perception [28]; furthermore, it is well known that skin characteristics such as response after insults and wound recovery deteriorate over the years [27].

Our results show that patients with PU compared to those without PU had significantly longer ICU stays between the event (sABI) and IRU admission, i.e., a longer time post event. Previous research findings concerning the length of stay in ICU and PUs were contradictory [30]. Actually, ICU stay implies limited movements and restricted positions, which are well-known major risk factors for PU onset [30], while stroke patients are at higher risk of immobility-related complications due to cognitive, functional, and sensory deficits [31]. A significant relationship between immobility and PU onset was reported, with a significant association between FIM score at admission and PU onset during hospitalization [31], confirming a protective role of rehabilitation to recover mobility and improve functionality. However, our data failed to observe a significant association between the FIM score and the presence of PU at admission or discharge.

The presence of malnutrition, which is reported in a high percentage of hospitalized patients [32], is a critical clinical issue in acute as well as rehabilitative settings because of its negative impact on several outcomes of interest. Specifically, malnutrition plays a central role in the onset of PUs which are in turn an independent predictor of unfavorable rehabilitation outcomes [13].

Nutritional deprivation and insufficient dietary intake are well-known PU risk factors [16]; for instance, underweight malnourished patients with low BMI have an increased risk of PU onset due to a multiplicity of risk factors such as the presence of skin areas with a bony prominence [30], in addition to decreased skin stretchability, reduced ability to fight against infections, impaired wound healing, etc. [16]. 

In addition, malnourished patients, who are often dehydrated, may have additional water needs if suffering from PUs due to the loss of fluid via injury exudate and sweating due to the use of pressure-relieving air mattresses [16].

However, in our study, at admission and discharge, the BMI of patients with PU when compared with that one of patients without PU showed no significant differences. 

In the present study, two tools for nutritional screening were used: MUST and CONUT. The MUST tool was designed to identify adults at nutritional risk in different settings [20]. The CONUT score was proposed for hospitalized patients, and it is considered as a nutritional inflammation index, first because of the impact of inflammation on albumin concentration.

In the study group evaluated, at admission to IRU the patients with PU exhibited a significantly higher MUST score (*p* = 0.008) (Table 1), whereas no significant difference emerged for CONUT. A possible explanation for these results may be that the onset of PU had mainly been related to decreased food intake due to the acute phase of the disease.

The MUST score includes the acute disease effect; as previously reported, all sABI patients, regardless of the etiology, are critically ill patients, characterized by very frequent comorbidities and medical complications.

Slightly different evidence emerged at the end of the rehabilitation period when 23 patients still presented PUs (Table 2). When compared to patients who had had their PU healed, patients with PUs were more often men and had a lower LCF score, suggesting a worst cognitive performance at admission. It is worth noting that the LCF scale is one of the most commonly used validated tools to assess the patient’s cognitive performance, not demanding patient collaboration [33,34]. The findings of the present study are consistent with those of previous studies showing that patients with low LCF scores at admission have a worse outcome [33]. Of note is that critically ill patients with impaired responsiveness and cognitive status are at high risk of PU onset due to impaired mobility and the absence of reaction to increased tissue pressure [28].

As for nutritional risk, the CONUT score at baseline was significantly higher in patients discharged with PU than in those who had their PUs healed and it was also identified as an independent negative predictor of PUs at discharge by a logistic regression model (Table 3). These findings are in line with previous evidence on the prognostic role of CONUT score in the short and long term, such as, for instance, in patients after stroke [35] or with heart failure [36] and in patients with ischemic wounds [23]. A possible explanation may be that the CONUT score includes laboratory parameters possibly associated with inflammatory status [35] and that chronic wounds are characterized by a prolonged inflammatory response [16]. Additionally, the CONUT score incorporating lymphocyte count and total cholesterol level reflects both the caloric intake and also the immune function which have both been a key role in wound healing.

The presence of PU at admission and discharge was associated with a higher inflammatory status, evaluated by NLR; however, no significant differences were observed between patients admitted and discharged with PU and those admitted and discharged without.

We can speculate that the CONUT score, assessing inflammatory and immunological parameters could be more helpful, in these critically ill patients, as a predictor of PU onset.

Another independent negative predictor of PUs at discharge is the PU severity at baseline (logistic regression model, Table 3). This finding is consistent with those of previous studies showing that the presence of PU is a risk factor for the potential progression of prior lesions [30] due to the complexity to heal severe lesions in patients highly vulnerable.

In this paper, we did not investigate the sABI subset malnutrition risk due to comparable nutritional and metabolic needs.

As previously reported, sABI patients are a heterogeneous group at higher malnutrition risk; studies report that patients with stroke or other critical neurological diseases have similar metabolic and nutritional needs compared to patients with traumatic brain injuries and comparable prevalence of malnutrition [9,37].

The main limitation of our retrospective and monocentric study is the lack of inclusion of other validate measures for assessing the risk of PU; however, a recent review [8] reports that PU risk-assessment tools are not effective in avoiding PUs onset.

Another limitation is that the study was not designed to investigate comorbidities, supplementary infections, and other risk factors of stress that can affect the nutritional and PU risk.

Furthermore, the present study was not designed to investigate inflammatory markers and PU prophylactic interventions/dressings. On the other hand, this study provides novel data on nutritional risk in a relatively large sample size of patients with sABI.

## 5. Conclusions

In terms of patients with severe acquired brain injuries (sABI), there is a lack of studies on the risk factors for pressure ulcer (PU) onset, and studies investigating the prognostic roles of nutritional tools are scarce.

Our results highlight the importance of assessing nutritional risk using simple standardized tools as part of the multidimensional evaluation of patients with severe acquired brain injuries (sABI) and pressure ulcers (PUs). The novelty of this paper is to show the association of nutritional risk with pressure ulcers in patients with severe acquired brain injuries; to the best of our knowledge, for the first time, the Controlling Nutritional Status (CONUT) score is demonstrated to be a negative predictor of pressure ulcer presence at discharge in patients with severe acquired brain injuries.

In this population, taking accurate weight and height measurements can be challenging [14] due to mobility impairment and bedridden status. In this regard, our results support the idea of assessing malnutrition risk not only with anthropometric parameters but also using simple laboratory tests, informative also on inflammatory and immunological patients’ status.

As previously reported, in these critically ill patients at high risk or with pressure ulcers, it is crucial for early classification of the nutritional status to define a tailored patient-focused nutrition plan. Additionally, in patients at risk or with pressure ulcers, it is important to consider specific supplements enriched with micronutrients to meet assessed needs and promote wound healing.

## Figures and Tables

**Table 1:** Clinical and demographic characteristics of patients with and without pressure ulcers at admission.

	No PU at Admission (*n* = 18) N (%) Median [IQR]	PU at Admission (*n* = 70) N (%) Median [IQR]	*p*-Value
Age	61 [15]	64 [20]	0.354
Sex (women)	4 (22.2%)	24 (34.3%)	0.327
BMI (kg/m^2^)	25.8 [3.6]	25.1 [6.6]	0.280
CRS at admission	12 [10.5]	14 [16.75]	0.233
LCF at admission	4 [2]	3 [2]	0.456
FOIS at admission	1 [0]	1 [0]	0.402
DRS at admission	19.5 [11]	21 [8]	0.176
FIM at admission	19 [9]	18 [3]	0.193
Etiology			0.637
Traumatic	5 (27.8%)	21 (30.0%)	
Hemorrhagic	4 (22.2%)	10 (14.3%)	
Ischemic	4 (22.2%)	13 (18.6%)	
Anoxic	3 (16.7%)	17 (24.3%)	
Other	2 (11.1%)	9 (12.9%)	
**Time post event (days)**	**30 [28]**	**44 [39]**	**0.034**
KCal tot	1800 [27.8]	1800 [426.8]	0.270
**MUST at admission**	**2 [0]**	**2 [2]**	**0.008**
CONUT at admission	5.5 [4.25]	5 [4]	0.145
Neutrophil/lymphocytes at admission	3.3 [3.6]	4 [4.1]	0.213

PU: pressure ulcers; CRS-R: Coma Recovery Scale-revised; LCF: Level of Cognitive Functioning Scale; FOIS: Functional Oral Intake Scale; DRS: Disability Rating Scale; FIM: Functional Independence Measure; MUST: Malnutrition Universal Screening Tool; CONUT: Controlling Nutritional status.

**Table 2:** Clinical and demographic characteristics of patients who had their PU healed or not during the rehabilitation period, at discharge.

	No PU at Discharge (*n* = 47) N (%) Median [IQR]	PU at Discharge (*n* = 23) N (%) Median [IQR]	*p*-Value
Age	65 [19]	63 [28]	0.920
**Sex (women)**	**21 (44.7%)**	**3 (13.0%)**	**0.009**
BMI (kg/m^2^) at admission	25 [6]	24 [8]	0.658
CRS at admission	16 [16]	10 [19]	0.106
**LCF at admission**	**3 [1]**	**2 [2]**	**0.042**
DRS at admission	21 [8]	23.5 [9]	0.128
FIM at admission	18 [3]	18 [2]	0.460
Etiology			0.732
Traumatic	15 (31.9%)	6 (26.1%)	
Hemorrhagic	5 (10.6%)	5 (21.7%)	
Ischemic	10 (21.3%)	3 (13.0%)	
Anoxic	12 (25.5%)	5 (21.7%)	
Other	5 (10.6%)	4 (17.4%)	
Time post event (days)	43 [58]	45 [32]	0.661
Length of stay (days)	157 [101]	170 [86]	0.761
**Ulcers severity (higher grade) at admission**	**2 [2]**	**3 [1]**	**0.005**
Kcal tot at admission	1800 [379]	1800 [505]	0.900
Arm circumference at admission	28 [5]	29 [5]	0.677
MUST at admission	2 [2]	2 [2]	0.624
**CONUT at admission**	**4 [3.5]**	**6 [6]**	**0.039**
Neutrophil/lymphocytes at admission	3.8 [2.9]	3.8 [4.8]	0.600

CRS-R: Coma Recovery Scale-revised; LCF: Level of Cognitive Functioning Scale; FOIS: Functional Oral Intake Scale; DRS: Disability Rating Scale; FIM: Functional Independence Measure; MUST: Malnutrition Universal Screening Tool; CONUT: Controlling Nutritional status.

**Table 3:** Association between the PU presence at discharge and clinical characteristics at admission. Logistic regression model (independent variables: demographic and clinical characteristics at admission; dependent variable: presence of pressure ulcers at discharge).

R^2^ = 0.351	B	SE	*p*-Value	OR	95% CI (OR)	
					Inferior	Superior
Sex	−1.308	0.773	0.090	0.270	0.059	1.229
LCF at admission	−0.505	0.273	0.064	0.603	0.353	1.030
**Ulcers severity (higher grade)**	**0.723**	**0.367**	**0.049**	**2.060**	**1.004**	**4.226**
**CONUT**	**0.312**	**0.146**	**0.032**	**1.366**	**1.027**	**1.818**

LCF: Level of Cognitive Functioning Scale; CONUT: Controlling Nutritional status (for 1 point).

## Data Availability

The data presented in this study are available on request from the corresponding author.
